# Lifespan Extension and Motor Function Improvement Effects of Whale Meat Extract in *Caenorhabditis elegans*

**DOI:** 10.3390/ijms252312833

**Published:** 2024-11-28

**Authors:** Junko Shibato, Fumiko Takenoya, Ai Kimura, Michio Yamashita, Randeep Rakwal, Seiji Shioda

**Affiliations:** 1 Department of Functional Morphology, Shonan University of Medical Sciences, 16-48 Kamishinano, Totsuka-ku, Yokohama 244-0806, Kanagawa, Japan; rjunko@nifty.com; 2 Department of Sport Sciences, School of Pharmacy and Pharmaceutical Sciences, Hoshi University, 2-4-41 Ebara, Shinagawa-ku, Tokyo 142-8501, Japan; kuki@hoshi.ac.jp (F.T.); francfranc.fragola@gmail.com (A.K.); d1902@hoshi.ac.jp (M.Y.); 3 Institute of Health and Sport Sciences, University of Tsukuba, 1-1-1 Tennodai, Tsukuba 305-8574, Ibaraki, Japan; plantproteomics@gmail.com

**Keywords:** cell culture, lifespan, gene expression, whale meat extract, imidazole peptide, *C. elegans*

## Abstract

The average life expectancy is increasing worldwide, but the same cannot be said for a healthy life expectancy (defined as the period during which a person can live a healthy and independent life). Therefore, a major challenge is how to extend healthy life expectancy. One option is to reduce age-related muscle atrophy (sarcopenia) and cognitive decline. Since there is no specific cure for frailty, the prevention of frailty based on nutrition and exercise is a new approach to achieve healthy longevity. Studies have shown that interventions combining nutritional supplements and exercise are effective in improving muscle strength, muscle mass, and walking speed. Thus, the search for drugs and functional foods to combat frailty has attracted researchers’ attention. Whale meat extract (WME) contains many imidazole dipeptides, especially the unique component balenine, which has various functional anti-fatigue and antioxidant properties, and hypermobility effects. Here, we investigated the effects of WME on the aging and health of *Caenorhabditis elegans* (hereafter, *C. elegans*). mRNA expression analysis showed that WME prolongs the lifespan of *C. elegans* mainly through *sir-2.1*, *daf-2*, and *daf-16*, and the *myo-3*, *unc-54*, *unc-22*, and *fhod-1* genes involved in locomotor function. The results of this study showed that the expression of the antioxidant enzymes *sod-2* and *sod-3* was also increased. This study may provide the basis for further research on WME as a food and supplement to slow aging and improve motor function.

## 1. Introduction

Healthy life expectancy is an indicator by the World Health Organization (WHO) that indicates the “average period of unrestricted daily life” during which a person can live independently and in good health. Efforts to narrow the gap between average life expectancy and healthy life expectancy are important, since even if life expectancy itself increases, it becomes less significant with declining health and mobility. Lifestyle modification, diet, and exercise are considered important to prevent the disease development and cognitive decline associated with aging and to extend healthy life expectancy. Recently, the intake of natural products has attracted attention as a simple way to reduce various risks associated with aging.

We have been conducting various investigations for the use of balenine [1,2,3], an imidazole dipeptide highly concentrated in whale meat, as an anti-fatigue food. It is becoming clear that imidazole dipeptide has antioxidant, anti-fatigue, and athletic performance-enhancing effects [4,5,6], improves cognitive function [7,8,9], and has a role in maintaining muscle function [10]. While carnosine and anserine have been studied variously, including their use as supplements to improve health, increasing research is being carried out on balenine, and it has been recently discovered that human skeletal muscle contains balenine [11]. Like carnosine and anserine, balenine has been shown to be effective in improving antioxidant, anti-fatigue, and memory functions [12,13]. However, balenine has higher antioxidant and iron-chelating effects compared to carnosine and anserine [12], and its stability in vivo is higher than that of carnosine and anserine [13], which has been suggested to be effective for use in ergogenic supplements.

Based on the results of studies showing increased muscle endurance in a ddY diabetic mouse model [14], the alleviation of fatigue due to high workload in humans [15], increased levels of learning and memory formation in SAMP8 mice [16], and the promotion of skeletal muscle regeneration [17] we hypothesized that whale meat extract (WME) could be used to prevent frailty. Thus, we investigated the effects of a WME-added diet on lifespan and exercise function via a molecular genetic approach using the free-living nematode *Caenorhabditis elegans* (*C. elegans*) as an animal model. The expression of the upstream (*daf-2* and *age-1*) and downstream (*daf-16*) regulators of insulin/IGF-1 signaling involved in lifespan, as well as muscle genes *myo-3* (MHC A), *unc-54* (MHC B), *unc-22*, and *fhod-1* involved in locomotion and body length were confirmed. In addition, the possible anti-aging effects of WME were explored by confirming the expression of the antioxidant enzymes *sod-2* and *sod-3*.

*C. elegans* exhibits muscle mass and sarcopenia comparable to human aging, with a gradual decline in body movement with age [18]. These characteristics make it a particularly well-suited and simple model organism for the study of a healthy lifespan. Elucidating the molecular mechanisms by WME may help to better understand the biological mechanisms associated with lifespan, as well as the behavioral consequences of age-related movement impairments.

## 2. Results and Discussion

### 2.1. Lifespan Assay

**Figure 1:**
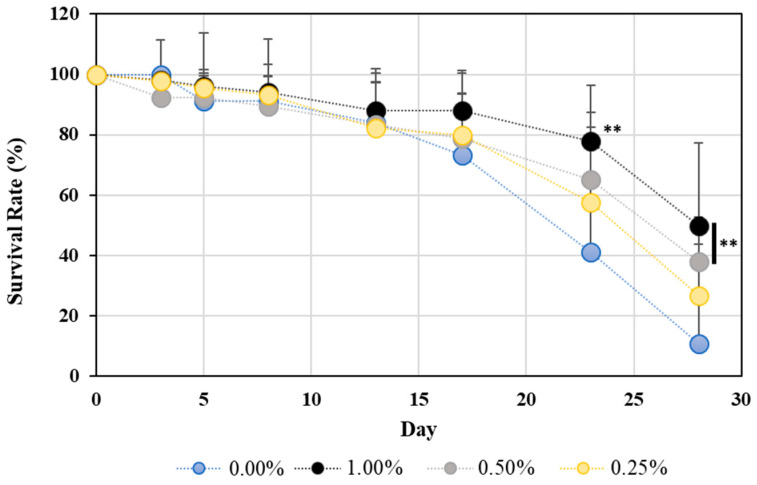
Lifespan extension effect of WME on *C. elegans*. The graph shows the survival rate, with the day of WME addition taken as 100%. Results are presented as mean ± SE ** *p* < 0.01 when compared to control (0.00% WME) values by Student’s *t* test.

The *C. elegans* longevity assay was performed three times independently, with the day of WME addition as day 0. Since almost the same results were obtained in all three experiments, the results of the first experiment are shown in Figure 1. The survival rate of *C. elegans* increased in proportion to the amount of WME added, and significant differences were observed under the conditions of 0.5% and 1.0% WME.

### 2.2. Motor Function Assay

#### 2.2.1. Flexion and Extension Movement Assay

**Figure 2:**
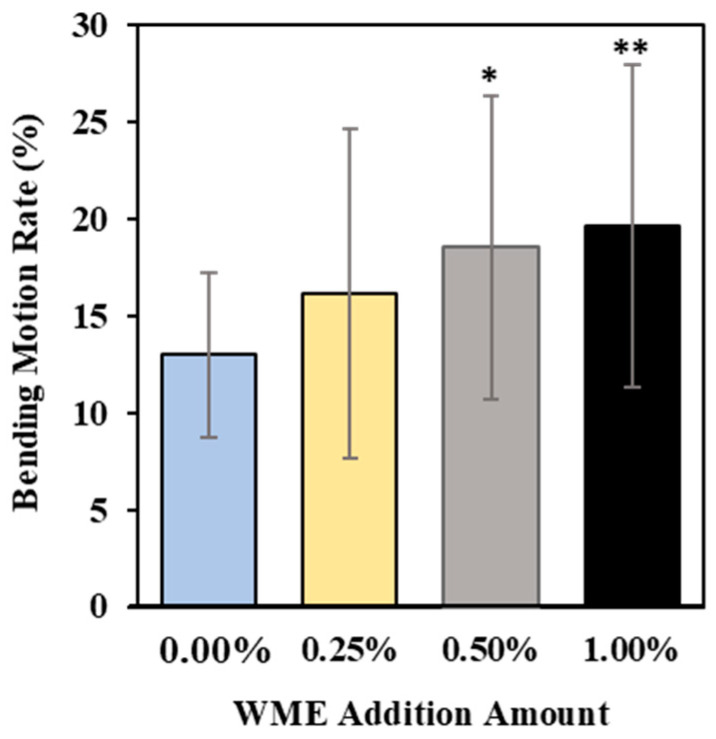
Refraction motility assay measurement results on day 3 after WME addition. Results are presented as mean ± SE ** *p* < 0.01, * *p* < 0.05 when compared to control (0.00% WME) values by Student’s *t* test.

The results of the flexion motor assay measurements on day 3 after the addition of WME are shown in Figure 2. It was confirmed that the rate of flexion motility increased in proportion to the amount of WME added. In particular, a significant difference was observed under the 1.0% (WME) addition condition.

#### 2.2.2. Edge Assay

**Figure 3:**
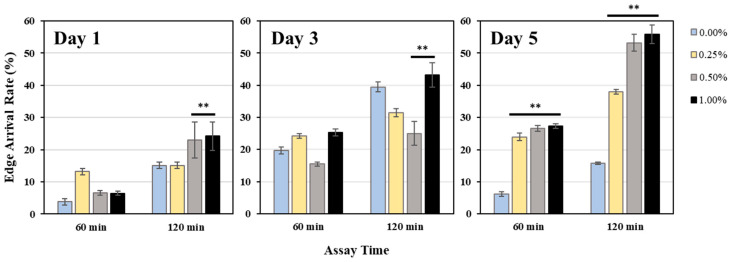
Edge assay measurement results on days 1, 3, and 5 after WME addition. The *E. coli* edge arrival rate after 60 and 120 min is shown. Results are presented as mean ± SE ** *p* < 0.01 when compared to control (0.00% WME) values by Student’s *t* test.

Figure 3 shows the results of edge assay measurements at 1, 3, and 5 days after the addition of WME. On the 5th day, the edge assay results showed a significant increase in the edge assay rate in proportion to the amount of WME added.

#### 2.2.3. Body Length Measurement

**Figure 4:**
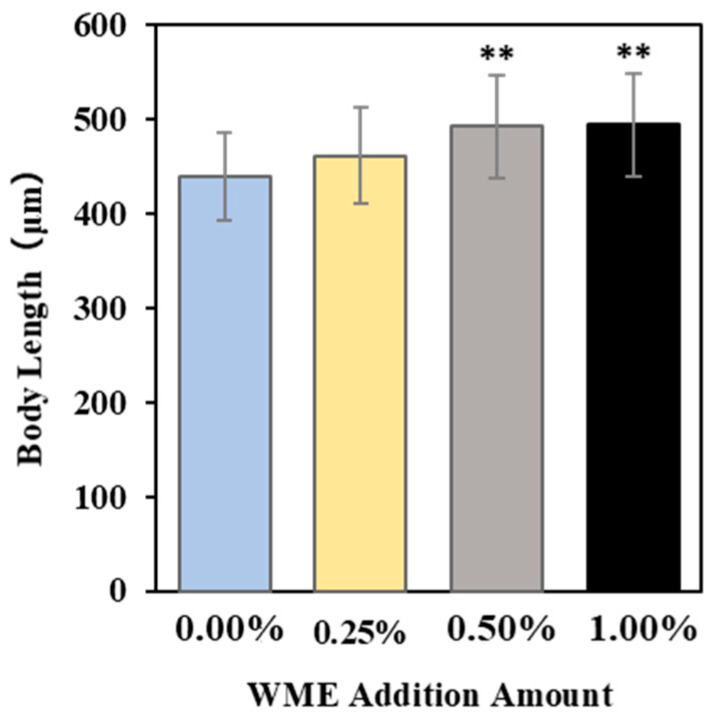
Measurement of body length (μm) of *C. elegans* 3 days after WME addition. Results are presented as mean ± SE ** *p* < 0.01 when compared to control (0.00% WME) values by Student’s *t* test.

Figure 4 shows the results of body length measurements of *C. elegans* on the third day after the addition of WME, and it was confirmed that body length increased in proportion to the amount of WME added. The results show that the body length of *C. elegans* increased in proportion to the amount of WME added, especially in the 0.5% and 1.0% addition conditions.

### 2.3. Gene Expression Analysis

**Figure 5:**
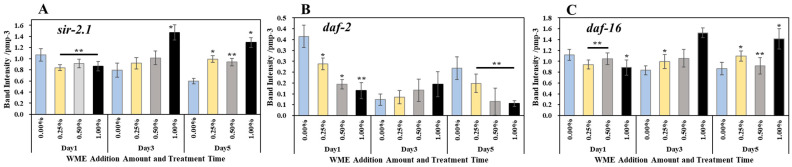
Lifespan-related *sir-2.1* (**A**), *daf-2* (**B**), and *daf-16* (**C**) gene expression results of *C. elegans* 1, 3, and 5 days after WME addition. Results are presented as mean ± SE ** *p* < 0.01, * *p* < 0.05 when compared to control (0.00% WME) values by Student’s *t* test.

**Figure 6:**
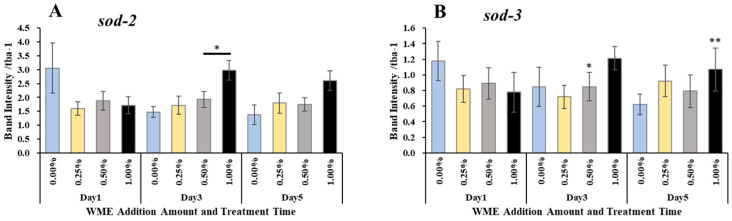
Antioxidant-related *sod-2* (**A**) and *sod-3* (**B**) gene expression results of *C. elegans* 1, 3, and 5 days after WME addition. Results are presented as mean ± SE ** *p* < 0.01, * *p* < 0.05 when compared to control (0.00% WME) values by Student’s *t* test.

**Figure 7:**
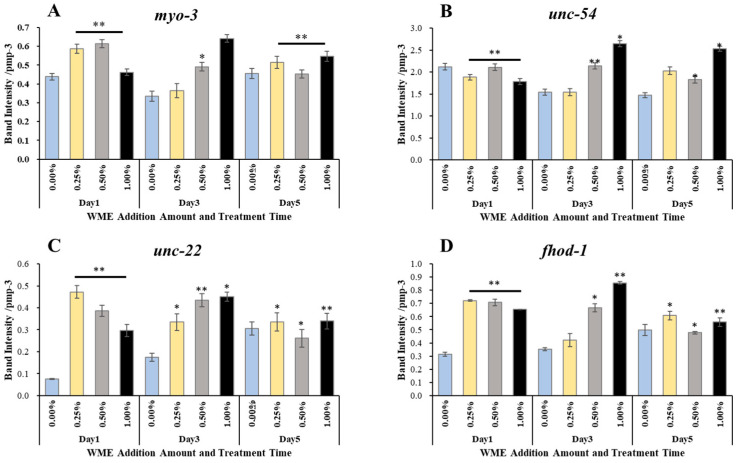
Muscle-related *myo-3* (**A**), *nuc-54* (**B**), *nuc-22* (**C**), *fhod-1* (**D**) gene expression results in *C. elegans* 1, 3, and 5 days after WME addition. Results are presented as mean ± SE ** *p* < 0.01, * *p* < 0.05 when compared with control (0.00% WME) values by Student’s *t* test.

RNA was extracted from *C. elegans* 1, 3, and 5 days after the addition of WME, and after cDNA synthesis, RT-PCR reactions using primers in Table 1 were used to analyze the expression levels of lifespan and muscle-related genes. The results are shown in Figure 5. In terms of lifespan-related genes, *sir-2.1* gene expression was significantly increased on day 1 for all 0.25, 0.5, and 1% (WME) additions. The expression of the *Daf-2* gene was significantly decreased in all 0.25, 0.5, and 1% (WME) additions on days 1 and 5 (Figure 5B). For the *daf-16* gene, a significant increase in expression was observed at the 0.25 and 0.5% (WME) additions on day 1 (Figure 5C). The expression of antioxidant enzymes *sod-2* and *sod-3* was highest in the control on day 1, but as the days passed, the expression of *sod-2* and *sod-3* in the control decreased (Figure 6A,B). On the other hand, with the addition of 1% WME, the expression level was lower than that of the control on day 1, but as the days progressed, the expression level increased (Figure 6A,B). The results for muscle-related genes are shown in Figure 7A–D. Overall, WME addition increased muscle-related gene expression. The *myo-3*, *unc-22*, and *fhod-1* gene expression increased markedly with WME addition on days 1 and 3 (Figure 7A–C). With *unc-54*, the expression decreased with the passage of days in the control, while it increased with the addition of 1% WME (Figure 7D).

## 3. Discussion

**Figure 8:**
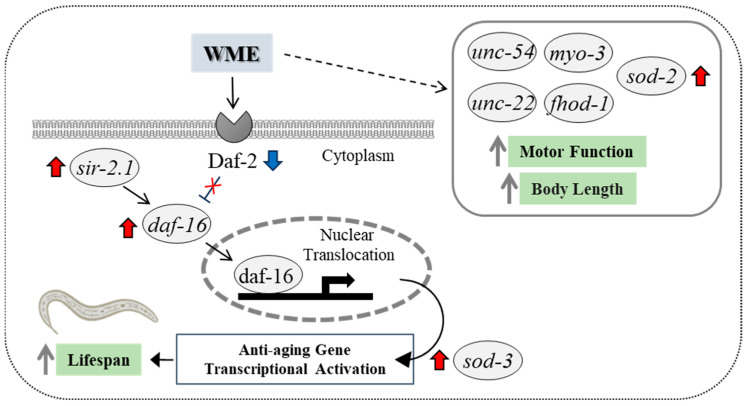
Scheme of WME-induced lifespan extension and motor function improvement in *C. elegans*. The figure depicts that under the present experimental conditions, WME improves lifespan and motor function through the upregulation (red arrows) of the *sir-2.1* and *daf-16* genes and the *sod-3* gene, as well as the *sod-2*, *myo-3*, *unc-54*, *unc-22*, and *fhod-1* genes; and, *daf-2* downregulation (blue arrow). However, there are other possibilities that have not yet been tested, such as other factors that regulate lifespan and motion capacity. These cannot be excluded in the current study.

The present experiments confirm the effects of WME on lifespan extension and locomotor function in *C. elegans*. The *daf-16* is required for lifespan extension in *C. elegans*, which is mediated by an increased expression of *sir-2.1* [19]. Furthermore, the only receptor to which insulin/IGF1 binds is *daf-2*, and when insulin/IGF1 binds to *daf-2* and activates the insulin/IGF1 signaling (IIS) pathway, the transcription factor DAF-16 remains in the cytosol, shortening lifespan. On the other hand, blocking the IIS pathway by inhibiting the *daf-2* function enhances the nuclear translocation of DAF-16, which in turn increases the expression of the anti-aging gene cluster and prolongs lifespan [20,21,22,23,24]. The results of the present study showed that the addition of WME suppressed the expression of the *daf-2* gene and increased the expression of the *daf-16* gene, suggesting that the lifespan-extending effect of WME is related to its anti-aging effects via *sir-2.1* and *daf-16* through *daf-2* suppression, as shown in Figure 8.

The *daf-16* gene is known to induce the expression of a number of genes encoding oxidative stress response enzymes, such as catalase (CAT) and sod-3 (superoxide dismutase, SOD) [25,26,27,28,29]. In the present experiment, we also observed the increased expression of *sod-2* and *sod-3* upon the addition of WME (Figure 6). Superoxide dismutase is an enzyme involved in detoxification, and SOD-related genes are involved in protection against oxidative stress in *C. elegans*. The *sod-3* gene encodes a *daf-16*-dependent SOD [30], and increased *sod-3* expression via *daf-16* is important for controlling oxidative stress and extending lifespan in *C. elegans* [25,31,32]. Increased damage during the aging process simultaneously triggers a protective superoxide response. The apparent increase in the body’s antioxidant enzymes, SOD activity, and CAT activity, which prolong lifespan, suggested that WME may have improved the antioxidant defense system in *C. elegans*. However, a lifespan extension was observed in strains deficient in the mitochondrial SOD gene, *sod-2*. This suggests that increased cytoplasmic reactive oxidative species (ROS) rather than increased mitochondrial ROS may be responsible for the short lifespan of *C. elegans* [33]. The *sod-2* and *sod-3* expression has been reported to increase lifespan [34,35,36], and mutations in *sod-2* or *sod-3* have been shown to suppress recovery from simulated microgravity toxicity. In addition, *sod-2* deletion mutants show developmental delay, a reduced number of offspring, and a defective excretory rhythm [37], *sod-2* has been implicated in *C. elegans* sperm pseudopodia elongation and sperm activation [38], and reduced *sod-2* and *sod-3* expression negatively affects locomotor behavior [39]. Considering these facts, *sod-2* expression is not essential for lifespan extension, but is important for antioxidant effects (SOD) and locomotor function improvement.

Muscle mass and sarcopenia are important features of aging, and patients with sarcopenia show a decrease in myofibrils. The *C. elegans* rhabdomere wall muscle is a simple model for sarcomere formation, as *C. elegans* migration is caused by the alternating contraction and relaxation of the dorsal and ventral rhabdomeres of the body wall; thus, the monitoring of muscle function is possible. The expression of many muscle genes in this experiment was increased by WME *myo-3*, *unc-54*, *unc-22*, and *fhod-1* (Figure 7), suggesting that these genes are involved in the improvement of motor function obtained by refractive exercise and edge assay. The *myo-3* and *unc-54* mRNA levels are reported to decrease with age [40,41]. In the present experiment, *nc-54* expression was progressively decreased with aging in controls, but not with the addition of WME (Figure 7B). The *unc-54* gene is essential for the maintenance of *C. elegans’* locomotion and sarcomere structural stability, and is also used to assess the *C. elegans* locomotor phenotype, and *myo-3* has also been suggested to be involved in body length [42]. The *fhod-1* gene has been implicated in rhabdomere development and strength in animals ranging from humans to *C. elegans*, and in *C. elegans*, *fhod-1* has been shown to promote sarcomere formation in the rhabdomeres and in *C. elegans*, and *unc-22* mutations are associated with slowed or paralyzed movement and disorganized muscular architecture in adults [43] and disorganized muscle segment structure [44].

## 4. Materials and Methods

### 4.1. C. elegans Maintenance Methods

*C. elegans* was maintained at 20 °C on *C. elegans* growth agar (NGM), spaced with OP50 *E. coli* cultured on LB medium Miller (Nacalai Tesque, Inc., Kyoto, Japan). The methods for medium and solution preparation followed the WormBook (http://www.wormbook.org/). The N2 strain (wild-type) of *C. elegans* was used (Gifted by Prof Mihira, Faculty of Medicine, Tokyo Women’s Medical University, Tokyo, Japan).

### 4.2. C. elegans Synchronization Method

Starved L1 worms on 5- to 10-day-old NGM plates were transferred to OP50-sprayed 100 mm NGM plates and maintained at 20 °C for 65 h. Gestation stage adults were rinsed from the NGM agar plates with sterile water and collected in 50 mL centrifuge tubes. Five mL of bleach solution was added, shaken well for a few seconds, and incubated for 5 min. Once the adults were observed to be dissolved, they were immediately neutralized with 5 mL M9 Buffer and centrifuged. The pellet of released eggs was washed three times with M9 Buffer and twice with S-basal before aspirating the supernatant and adding 40 mL of S-basal. After incubation overnight at 20 °C and 180 rpm, the number of hatched L1 stage larvae in 10 μL was determined and used for testing.

### 4.3. Adjustment of WME

WME (Balenine B8: Kyodo Senpaku Co., Ltd., Tokyo, Japan) is sold as a food, and its standard ingredients and total content are more than 9% imidazole dipeptide, more than 8% balenine, and more than 65% total amino acids. First, 10 g of balenine B8 was mixed with distilled water to make 10 mL. The sample was sterilized by passing it through a 0.22 μm filter after dissolution by vortex mixing.

### 4.4. Lifespan Assay

*C. elegans* was adjusted with S-medium at 8–15 worms/100 μL for the number of L1 stage larvae obtained and synchronously treated by hypochlorite bleaching. The food, *E. coli* OP50, was suspended in S-complete, added at 6.1 mg/mL, and incubated at 20 °C, 110 rpm. Two days (~44 h) after the addition of *E. coli*, *C. elegans* was washed with sterile water using a 40 μm filter, collected, and dispensed into 96-well plates at 5–12 fish/100 μL/well. At a final concentration of 120 μM FUDR (5-fluoro-2-deoxyuridine) (Sigma, St. Louis, MO, USA), dead *E. coli* OP50 was heat treated at 75 °C for 30 min (*E. coli* OP50 was heat treated at 75 °C for 30 min to minimize the effect of WME on *E. coli* and confirmed dead). In addition, WME was added at 0.00% (control), 0.25%, 0.50%, and 1.00% WME; the day of WME addition was day 0, and the number of days until all *C. elegans* died was observed. Incubations were conducted at 20–21 °C. Sixty to one-hundred *C. elegans* were used for each condition, and the average lifespan was calculated. Three independent experiments were conducted to confirm the lifespan effect; n = 3, approximately 180–300 animals. In each experiment, death was determined by the lack of response to contact with the platinum wire. Unexpected deaths due to straying or wall climbing were excluded. Statistical analysis of the data was performed using the *t*-test.

### 4.5. Motor Function Assay

As in the lifespan assay, dead *E. coli* OP50 mixed S-medium containing WME and a final concentration of 120 μM FUDR was added to *C. elegans* that were washed with sterile water using 40 μm filters and collected 2 days (~44 h) after the addition of *E. coli* (0, 0.25, 0.50, and 1.00%). The day of WME addition was day 0, and the flexion movement assay, edge assay, and body length measurement experiments were performed on day 3.

#### 4.5.1. Measurement of Bending Movement Frequency

Ten to fifteen *C. elegans* were transferred to NGM plates with 1 drop of M9 Buffer, and the number of animals that bent their bodies at least once over a 30 s period was calculated by microscopic observation (n = 12, using 3–14 animals each for a total of 80–104 animals).

#### 4.5.2. Edge Assay

Edge assay plates were prepared by 100 mm NGM agar plates; NGM plates were air-dried on a clean bench and kept at 4 °C until use. The day before the edge assay, sterile cotton swabs were soaked with *E. coli* suspension and the NGM plate was rotated 360° to spread *E. coli* around the entire edge of the plate. The plates were incubated overnight at 20 °C and used the next day. Synchronized *C. elegans* were collected, *C. elegans* washed with M9 Buffer were placed in the center of the edge assay plate, and excess M9 Buffer was removed with a Kimwipe. The number of *C. elegans* that reached or did not reach the edge was counted at 60 and 120 min to determine the percent reaching the *E. coli* edge. Edge assays were performed 1, 3, and 5 days after the addition of WME (n = 5, calculated using 10–30 animals each for a total of 50–150 animals).

#### 4.5.3. Measurement of *C. elegans* Body Length

Three days after the addition of WME, *C. elegans* body length was measured by microscopy and ImageJ ([https://imagej.net/ij/], accessed on 1 March 2024) image processing software (n = 3, using 10 animals each).

### 4.6. RNA Extraction for Gene Expression Analysis

As in the lifespan assay, dead *E. coli* OP50 mixed S-medium containing WME and a final concentration of 120 μM FUDR was added to *C. elegans* that were collected 2 days (~44 h) after the addition of *E. coli* and washed with sterile water using 40 μm filters. Day 0 was the day of WME addition and *C. elegans* were collected on days 1, 3, and 5, passed through a 40 μm mesh filter, washed with M9 Buffer, and then collected by centrifugation to pellet. *C. elegans* pellets were crushed by grinding in liquid nitrogen using a mortar, mixed with QIAzol Lysis Reagent (Qiagen, Hilden, Germany), and then stored at −80 °C until RNA extraction. The stored pellets were frozen and thawed three times, and total RNA was extracted according to the protocol of the RNeasy Mini Kit (Qiagen). Extracted RNA was measured for RNA concentration using a micro-spectrophotometer (DS-11, DeNovix, Wilmington, DE, USA), and the 260/280 and 260/230 ratios were confirmed to be greater than 1.8. More than 1000 animals were used for each total RNA extraction in this experiment.

### 4.7. RT-PCR Protocol

After synthesizing cDNA using the Affinity Script QPCR cDNA synthesis kit (Agilent, Santa Clara, CA, USA), 100 to 500 ng of RNA was subjected to PCR reactions using specific primers (Table 1) for the gene of interest using Emerald Amp PCR Master (Takara, Kusatsu, Japan). PCR reactions (initial denaturation at 97 °C for 5 min, thermal denaturation at 95 °C for 45 s, annealing at 55 °C for 45 s, extension at 72 °C for 1 min, 27 to 36 cycles, and extension at 72 °C for 10 min) were performed. After PCR reactions, the PCR products were separated on 1.5% agarose gels and visualized with ethidium bromide under UV light. The expression levels of the visualized target genes were corrected for pmp-3 gene expression, a known housekeeping gene, and graphed.

## 5. Conclusions

This study based on rigorous methodology and mRNA expression analysis indicates that certain aging pathways may be activated; it is difficult to formally conclude that the observed activity is responsible for the lifespan extension. Nevertheless, this is also the first experiment to confirm the lifespan-extending effect of WME with a high balenine content, and we believe that we can offer the possibility that *sir-2.1*, *daf-2*, and *daf-16* genes are involved as factors influencing this lifespan-extending effect. And, the antioxidant effects via the *sod-3* genes, as well as increased *sod-2*, *myo-3*, *unc-54*, *unc-22*, and *fhod-1* expression by WME, are involved in improving exercise function. Saying that, we have not tested other gene candidates or performed a full ’omics-wide’ study to screen potentially new molecular factors, genes, proteins, and metabolites, which should be a target of the next research. Our results highlight the potential of WME to ameliorate age-related decline, promote longevity, and further extend a healthy lifespan. Another limitation is that the study did not look at the long-term effects of WME at 10, 20, or 30 days (aged animals). It should also be noted that in this particular experiment, the WME powder was used, which is characterized by a total imidazole dipeptide of more than 9%, of which more than 8% is balenine. However, in future experiments, we would like to consider the use of purified balenine under the possibility of obtaining it at a reasonable price (cost is a factor limiting the use of purified balenine). Finally, we would like to add that we truly believe and hope that it will be understood that this research is not aimed solely at promoting overfishing, but is fundamental research aimed at adding value to the WME as a fishery resource and utilizing it without waste.

## Figures and Tables

**Table 1:** Primer design of genes used in RT-PCR analysis.

	Forward Primer	Reverse Primer	
Accession (Gene)	Nucleotide Sequence (5′-3′)	Nucleotide Sequence (5′-3′)	Gene Name
NM_001269678	agttgaatgggattggatgaag	ctatcgaggctgtgtcaatgtc	*pmp-3*
NM_001268555	ttgaaactggttcgtgatgttc	gtttttcaatgggatttggtgt	*sir-2.1*
NM_065249	atgagcatcatccacttgtctg	gtcagcgaacgtacaaactcag	*daf-2*
AF032112	ttcaatcgtgtggaattgtagc	tgggatgaatgtgttggaatta	*daf-16*
NM_059889	ctggactaatttggcaaaggac	agtagtaagcgtgctcccagac	*sod-2*
NM_078363	agaaccttcaaaggagctgatg	ctgcttttattgtcgagcattg	*sod-3*
NM_073664	ttaatgctcacgtgtctgctct	acgtctctgttctccatccaat	*myo-3*
NM_061195	gaatctgaattggacggagaac	ggagaaagagcatgtagggatg	*unc-54*
NM_001313484	agacctggtggacactgaatct	gagtgagtgaggaggaaggaga	*fhod-1*
NM_069872	aagctagagtgcaaggaacacc	cttaaaggtgtttccggtcttg	*unc-22*

## Data Availability

Data are contained within the article.
